# Scale‐Up Strategy Focused on Hydrodynamic Stress for Mammalian Cell Culture Established by a Dry‐Wet Approach

**DOI:** 10.1002/elsc.70054

**Published:** 2025-11-21

**Authors:** Hiroyuki Kenmoku, Akira Kaneko, Takanobu Saito, Takahiro Nemoto, Yoshikazu Kato, Shunsuke Ohira

**Affiliations:** ^1^ Upstream Bioprocess Science, Chemical and Biological Technology Laboratory, CMC Development, Astellas Pharma. Inc Ibaraki Japan; ^2^ Research & Development Section, Mixing Laboratory, SATAKE MultiMix Corporation Toda‐shi Saitama Japan

**Keywords:** bioreactor, CFD, CHO cell, scale up

## Abstract

Today, most recombinant protein drugs are produced by mammalian cells in a stirred‐type bioreactor (BR). Although cell culture scale‐up strategies have been extensively investigated, scale‐up and switching BRs while maintaining comparable culture performance remains a challenging step. This is because the empirical correlations used to determine operating parameters are applicable only for limited situations using similar BRs across scales. In addition, a few small scale‐down models (SSDMs) are able to evaluate cellular sensitivity to the shear environment of manufacturing‐scale BRs. In this study, we focused on the hydrodynamic stress associated with agitation and developed an SSDM that generates high shear stress without undesirable secondary effects such as vortex formation and severe gas hold‐up. In‐house BRs with various scales and configurations were used for fed‐batch culture of CHO‐K1 cells, and their shear environment was characterized by computational fluid dynamics (CFD). Using the dry‐wet approach, we found that average shear stress was well correlated with titer decrease as an indicator of culture performance. We also confirmed that the response to shear stress differs among cell lines, and that evaluation of the shear sensitivity of cells is accordingly a risk mitigation step that is required to ensure successful scale‐up.

**Abbreviations**
BR: bioreactor
CFD: computational fluid dynamics
CHO: Chinese hamster ovary
CMC: chemistry, manufacturing and control
DO: dissolved oxygen
EDR: energy dissipation rate
Fab: fragment monoclonal antibody
GBR: glass bioreactor
GEV: gas entrance velocity
mAb: monoclonal antibody
*P*/*V*
: power input per unit volume
PP: pitched paddle
RANS: Reynolds‐averaged Navier Stokes
SSB: stainless steel bioreactor
SSDM: small scale‐down model
SUB: single‐use bioreactor
VOF: volume of fluid

## 1 Introduction

The development timeline of biopharmaceuticals such as monoclonal antibodies (mAb) is accelerating, providing faster access to patients [1, 2]. Recombinant protein drugs are produced by microorganisms or mammalian cells, most commonly Chinese hamster ovary (CHO) cells, and titers of over 10 g/L have been achieved in a fed‐batch culture [3, 4]. Progress in upstream bioprocess science such as high titer culture media, intensified culture methods and gene‐edited host cell lines with site‐specific integration technology enable the time required for chemistry, manufacturing and control (CMC) activities to be minimized, including clone selection and process development [5, 6, 7].

Recently, single‐use bioreactors (SUBs) have been widely used for 1–2 kL scale cell culture [8]. Various types of SUBs are available from vendors, which generally ensure the scalability of SUBs from lab‐ to manufacturing‐scale [9, 10]. Scale‐up within the same type of SUB is commonly performed based on empirical correlations, such as a consistent power input per unit volume (*P*/*V*), tip‐speed, mixing time, gas‐flow rate, and oxygen mass transfer [11, 12, 13]. However, when it comes to the scale‐up and scale‐down among different configurations of bioreactors (BRs), the use of empirical correlations to determine BR operating conditions remains difficult. Interestingly, one study reported the interchangeable use of two different SUBs, with a focus on sparger design [14].

Because of accelerated timelines and limited manufacturing capacity, it is not practical to utilize the same scale and type of BR throughout product development, and technology transfer across scales and even BR types is often considered. For the commercial stage, further scale‐up from SUBs to 5‐ to 10‐fold volume steel use stainless bioreactors (SSBs) is one option for accommodating increased product demand. However, there are a number of difficulties in carrying out further scale‐up from SUBs owing to the lack of empirical or theoretical knowledge about the determination of agitation rate and gassing strategy across scales. Additionally, it remains challenging to establish a small scale‐down model (SSDM), which reproduces the stress environment of manufacturing‐scale BRs, in which higher shear stress can be generated. Further, the adaptability of cell lines for such severe culture conditions is not typically evaluated during clone selection and process development. These background factors, therefore, mandate a deep understanding of the characteristics of BRs and identification of a factor that both reflects the stress environment and can be applied across non‐similar BRs.

Today, computational fluid dynamics (CFD) is widely recognized as a useful tool for understanding the characteristics of BRs. The ability to visualize fluid flow patterns in a BR by CFD in turn allows the culture environment, such as the distribution of shear stress, energy dissipation rate (EDR), and flow velocity, to also be quantified [15, 16]. This information enables comparison of the stress environment across any scale and type of BR. Indeed, CFD has been used to characterize not only manufacturing‐scale but also lab‐scale BRs, and successful use in optimizing operating conditions, such as agitation rate, has been reported [17, 18, 19, 20, 21, 22, 23]. Single liquid‐phase and multi (typically gas and liquid)‐phase CFD methods are widely used. A volume of fluid (VOF) method is often used to trace movement of the gas–liquid surface of cell culture [16, 24]. It is important to select an appropriate CFD model based on the objectives and conditions of the analysis. Generally, the Reynolds‐Averaged Navier‐Stokes (RANS) model is recognized for its computational efficiency and practicality, although it has certain limitations in accuracy. In contrast, direct numerical simulation (DNS) provides high precision at a substantial computational cost, while large eddy simulation (LES), although still costly, offers a balanced approach between the two [16].

Because mammalian cells were previously thought to be shear‐sensitive due to their lack of a cell wall, researchers paid major attention to BR design and culture conditions [12, 25]. Extensive studies of the effect of hydrodynamic stress on mammalian cells in suspension culture have suggested that the source of shear damage to cells is mainly attributable to three mechanisms: (1) physical damage induced by impeller agitation, (2) damage directly caused by gas bubble rupture, and (3) damage associated with gas entrance velocity (GEV) through the sparger at entry into the culture [26]. However, thanks to the successful use of CHO cells in manufacturing‐scale suspension culture to produce mAb therapeutics, it is now also thought that shear sensitivity under current “normal” culture conditions is no longer a major problem [27].

High shear stress induced by agitation is observed at the interface of liquid to vessel wall and near structures such as impellers and baffles. Increasing agitation rate is one option to achieving homogeneous mixing and providing sufficient oxygen and removal of CO_2_ from culture. Several studies concluded that “normal” operating agitation rates—including agitation up to 600 rpm in a 2 L BR [28] and a *P*/*V* of 10–100 W/m^3^ at various production scales [29]—have little impact on the performance of suspension cell cultures. Neunstoecklin et al. experimentally determined thresholds for the maximum tolerable hydrodynamic stress of 25.2 ± 2.4 and 32.4 ± 4.4 Pa for mouse hybridoma Sp2/0 and CHO cells, respectively, using a 3 L BR and the oscillating stress loop system [30]. They further verified thresholds for Sp2/0 cells with a 300 L pilot scale BR [31].

The effects of hydrodynamic stress are often discussed as being lethal. Conversely, there are few reports evaluating hydrodynamic stress as a sub‐lethal effect, such as its propensity to reduce productivity. A meta‐analysis conducted by Chalmers compared previous studies by using the average EDR as an indicator of hydrodynamic stress and found that it ranged from 10^1^ to 10^9^ W/m^3^. The reported cellular responses include lethal responses, such as apoptosis and necrosis, primarily observed at 10^6^–10^8^ W/m^3^, and sublethal responses, such as reduced productivity and metabolic changes without severe viability decrease, observed in the lower range [27]. However, some of these studies only utilized micro or bench‐top scale BRs, which may require high agitation rates to generate high hydrodynamic stresses, potentially leading to vortex formation and gas hold‐up. Therefore, it could be said that these previous studies did not necessarily focus on practical evaluations and applications that address challenges such as scale‐up, scale‐down, and changes in BRs. Since a decrease in productivity has a business impact, namely an increase in manufacturing cost per unit produced, it is important to investigate the sub‐lethal effects of hydrodynamic stress. As an example of scale‐up across scales and types of BRs using CFD, one report described successful scale‐up from a 2 kL SUB to a 10 kL SSB based on mixing time and volumetric oxygen mass transfer coefficient (kLa) [21]. It is also suggested that scale‐up strategies based on classical empirical rules that combine *P*/*V* and gas‐flow rate, or by setting operating conditions based on the kLa ratio (kLa [CO_2_]/kLa [O_2_]), are helpful in certain conditions [32, 33].

In this study, we focused on hydrodynamic stress in BRs and developed an SSDM that can generate high shear environment without causing undesirable secondary effects such as vortex formation and gas hold‐up. In‐house BRs of various sizes and configurations were used for fed‐batch culture of CHO‐K1 cells at several agitation rates, and their shear environment was characterized by CFD. Multivariate analysis was then performed to identify correlations between parameters reflecting the culture environment and titer decrease as an indicator of culture performance. Additionally, to determine whether sensitivity to shear stress was dependent on cell line, several cell lines were cultured under multiple different stress conditions to evaluate their stress tolerance.

## 2 Materials and Methods

### 2.1 Bioreactor Configurations

**FIGURE 1:**
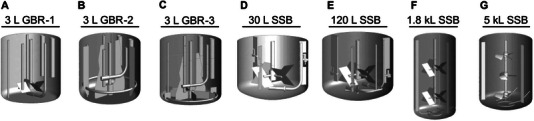
Bioreactor design. GBR indicates glass bioreactor; SSB, stainless steel bioreactor.

A total of seven BRs were evaluated (Figure 1 and Table 1). The 3 L glass bioreactor (GBR)‐1 (ABLE, Japan) has a four‐pitched paddle (4PP) impeller. The 3 L GBR‐2 has a special large and wide type impeller named MR210Bio (Satake Multimix, Japan). The 3 L GBR‐3 is equipped with an MR216Bio and six wide stationary blades as baffles (Satake Multimix, Japan). The MR216Bio consists of an MR210Bio impeller and four side impellers which help generate high shear stress without forming a vortex. All 3 L GBRs have a 120 µm frit sparger. The 30 and 120 L SSBs have a 4PP impeller with three baffles and 100 µm frit sparger (s). The 1.8 kL SSB is equipped with 2 × 4 PP impellers with three baffles and the 5 kL SSB is installed with 3 × 3 axial flow (AF) impellers with three baffles. All SSBs used in this study were designed for and utilized in‐house BRs. They all differed with regard to geometry, such as impeller design and baffle conditions.

**TABLE 1:** Bioreactor configuration

#	Bioreactor	Impeller	No. of baffles	*d*/*D*	*H*/*D*	Typical working volume (kg)	Maximum working volume (kg)	Sparger type and average pore size
A	3 L GBR‐1	1 × 4PP	None	0.50	0.92	1.5	2.0	1 × 120 µm Frit
B (B‐1)	3 L GBR‐2	1 × MR210Bio	3	0.74	1.00	1.5	2.0	1 × 120 µm Frit
C (B‐6)	3 L GBR‐3	1 × MR216Bio	6 ^a^	0.74	1.00	1.5	2.0	1 × 120 µm Frit
D	30 L SSB	1 × 4PP	3	0.66	0.99	25	30	1 × 100 µm Frit
E	120 L SSB	1 × 4PP	3	0.60	1.03	100	120	2 × 100 µm Frit
F	1.8 kL SSB	2 × 4PP	3	0.63	2.15	1600	1800	DHS and Frit
G	5 kL SSB	3 × 3AF	3	0.41	1.43	4000	5000	DHS and Frit

Abbreviations: AF, axial flow impeller; *d*/*D*, impeller diameter per bioreactor diameter; DHS, drilled‐hole sparger; GBR, glass bioreactor; *H*/*D*, height at maximum working volume per bioreactor diameter; PP, pitched paddle; SSB, stainless steel bioreactor.

^a^ Six fixed blades with a height of 32 mm are installed as baffles.

### 2.2 CFD Simulation

CFD simulation was conducted using ANSYS 19.2 (ANSYS Inc., USA) to visualize and quantify the culture environment of BRs. Shear stress, EDR, Kolmogorov scale, *P*/*V*, and flow velocity were simulated. The mesh used for CFD was created using ANSYS ICEM CFD 19.2 (ANSYS Inc., USA). The mesh size was determined based on prior internal knowledge, resulting in approximately 2–5 million tetrahedral meshes, which has been confirmed to be sufficient for accurately reproducing the internal shape of the BRs. CFD was implemented with a steady‐state analysis and the RANS approach, specifically the realizable k‐ε model. We internally validated that the fluid flow within a BR can be adequately simulated using this RANS method. A liquid single‐phase method was applied for the 30 and the 120 L SSBs. For the other BRs, a liquid–gas two‐phase method was used with a VOF method to correctly describe the free liquid surface, especially under high agitation rate conditions. The analysis settings for the CFD software are based on comparisons with past internal experiments, and the settings are such that the power and flow velocity are within 5% of the actual measurements.

Shear stress *τ* (Pa) is the force per unit area acting tangentially to the fluid flow, and is expressed as:

(1) τ=μγ

where μ (Pa s) is the effective viscosity, expressed as:

(2) $$\mu =\mu_{\mathrm{mole}}+\mu_{\mathrm{turb}}$$

where *µ*_mole_ is the molecular viscosity and *µ*_turb_ is the turbulent viscosity. The shear strain rate *γ* (s^−1^) is expressed using the velocity gradient in the fluid as:

(3) $$\gamma =\sqrt{\begin{array}{c} 2\left[{\left(\frac{\partial u}{\partial x}\right)}^{2}+{\left(\frac{\partial v}{\partial y}\right)}^{2}+{\left(\frac{\partial w}{\partial z}\right)}^{2}\right]+{\left(\frac{\partial u}{\partial y}+\frac{\partial v}{\partial x}\right)}^{2}\\ \;+{\left(\frac{\partial v}{\partial z}+\frac{\partial w}{\partial y}\right)}^{2}+{\left(\frac{\partial w}{\partial x}+\frac{\partial u}{\partial z}\right)}^{2} \end{array}}\;$$

The EDR *ε* (m^2^/s^3^) is the dissipation rate of turbulent kinetic energy *k* (m^2^/s^2^) and is obtained by solving the following transport equation:

(4) $$\begin{array}{ccc} \frac{\partial \left(\rho \varepsilon \right)}{\partial t}+\frac{\partial \left(\rho \bar{U_{i}}\varepsilon \right)}{\partial x_{i}} & = & \frac{\partial }{\partial x_{i}}\;\left\{\left(\mu +\frac{\mu_{t}}{\sigma_{\varepsilon }}\right)\frac{\partial \varepsilon }{\partial x_{i}}\right\}\\ & & +\rho C_{1}S_{\varepsilon }-\rho C_{2}\frac{\varepsilon^{2}}{k+\sqrt{\mu \varepsilon }} \end{array}$$

where *ρ* is the density and $\bar{U_{i}}$ is the Reynold‐averaged velocity. Kolmogorov scale *λ* (m) is the smallest length scale in the fluid and is expressed as:

(5) $$\lambda ={\left(\frac{{\left(\mu_{\mathrm{mole}}/\rho \right)}^{3}}{\varepsilon }\right)}^{\frac{1}{4}}\;$$

*P*/*V* (W/m^3^) is calculated using the agitation rate *n* (s^−1^), torque value *T* (N m), and fluid volume *V* (m^3^) as follows:

(6) $$P/V=\frac{2\pi nT}{V}\;$$

The average value was calculated by dividing the sum of the products of the scalar quantity in the element and its element volume by the total volume, and if the scalar quantity is *φ_i_*, it is expressed as:

(7) $$\frac{1}{V}\sum_{i=1}^{n}\varphi_{i}\left|V_{i}\right|$$

The maximum value is the node value of the element with the largest scalar quantity among all the volume elements in the analysis domain.

The effect of aeration from spargers was ignored because the aeration rate during cell culture was low, and also because the culture medium contained the same poloxamer concentrations commonly used in cell culture to prevent cell damage due to bubble rupture at liquid surface. CFD analyses were employed at three levels of tip‐speed for each BR unless otherwise noted.

### 2.3 Cell Lines and Media

A single in‐house recombinant CHO cell line (Cell line A), which expresses mAb A was used for all studies. For clone selection study, six mAb A‐expressing in‐house recombinant CHO cell lines (clone A, B, C, D, E, and F) and 12 fragment monoclonal antibody (Fab) A‐expressing in‐house recombinant CHO cell lines (clone a, b, c, d, e, f, g, h, i, j, k, and l) were used. Note that cell line A and clone C are the same cell line. All cell lines were established using CHO‐K1 cells as host (ATCC #CCL‐61) and were selected in CD CHO medium (Thermo Fisher Scientific, Waltham, MA, USA) containing L‐methionine sulfoximine. A chemically defined in‐house basal medium and feed media were used for the fed‐batch culture process. The in‐house basal medium contained the same poloxamer concentrations as those commonly used in cell culture to prevent cell damage due to bubble rupture at liquid surface.

### 2.4 Cell Culture

Production culture was run in fed‐batch mode using 3 L GBRs, a 30 L and a 120 L SSB. All production cultures were initiated at 1.0 × 10^6^ cells/mL of initial viable cell density (VCD) with a 1.2–1.5 kg working volume for the 3 L GBRs and a 20 and 100 kg working volume for the 30 and 120 L SSB, respectively. During cell culture, two feed media were added at certain time points at a fixed percentage, and glucose and antifoam were added as required. pH was controlled at 7.00 ± 0.30 with CO_2_ gas and 1N NaOH. Dissolved oxygen (DO) was maintained above 3.0 ppm using a combination of air and O_2_ gas. To mitigate CO_2_ accumulation in cell culture, CO_2_ stripping was conducted by air sparge based on the partial pressure of CO_2_ (*p*CO_2_). Agitation rate was determined considering the average shear stress simulated by CFD. To monitor the cell culture profile, 10 mL of culture fluid was aseptically taken from the bioreactor using a syringe every day from inoculation to harvest. For harvesting, prior to the end of the production culture, 50 mL of culture fluid was aseptically taken from the bioreactor using a syringe and centrifuged at 3000 rpm for 15 min at room temperature. VCD, viability, and cell size were measured by the trypan blue exclusion method using a Vi‐CELL XR (Beckman Coulter, Brea, CA, USA). DO and pH were monitored using in‐line probes installed in the BRs (Mettler Toledo, Switzerland). Glucose, lactate, ammonium, and *p*CO_2_ were determined using Bio Profile400 (Nova Biomedical, Waltham, MA, USA). mAb titer and Fab titer were measured using a protein A affinity column (Thermo Fisher Scientific, Waltham, MA, USA) or protein L affinity column (Thermo Fisher Scientific, Waltham, MA, USA), respectively, and an HPLC system (Agilent, Santa Clara, CA, USA) with a UV detector.

## 3 Results and Discussion

### 3.1 Hydrometric Characterization

**FIGURE 2:**
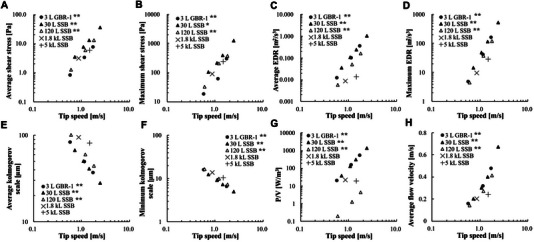
Regression between CFD output and tip‐speed. EDR indicates energy dissipation rate; GBR, glass bioreactor; *P*/*V*, power input per volume; SSB, stainless steel bioreactor.

Figure 2 shows relationships between CFD outputs (average and maximum shear stress, average and maximum EDR, average and minimum Kolmogorov scale, *P*/*V*, and average flow velocity) and tip‐speed. Coefficients of determination (*R* ^2^) for all power approximations were > 0.95, indicating the accuracy of the regression model is sufficient. The correlation between CFD output and tip‐speed will likely be useful in estimating these parameters at other tip‐speeds without additional CFD simulation, and further identification of a scale‐up factor related to hydrodynamic stress by agitation. Of note, no significant difference in CFD output was observed between the VOF method and the single‐phase method (data not shown).

### 3.2 Development of a Small Scale‐Down Model That Can Reproduce High Shear Stress

The CFD results indicated that average shear stress in manufacturing‐scale BRs was up to 6 Pa (3.02 Pa in the 1.8 kL SSB [tip‐speed: 0.86 m/s] and 5.85 Pa in the 5 kL SSB [tip‐speed: 1.47 m/s]), while average shear stress in the 3 L GBR‐1 was 0.85–7.83 Pa at a tip‐speed of 0.57–1.70 m/s. Another CFD analysis found that the average shear stress of the 1–2 kL SUBs was also up to 6 Pa with the recommended tip‐speed condition for typical CHO cell culture (data not shown). The 3 L GBR‐1 reproduced the average shear stress in manufacturing‐scale BRs with an extremely high agitation rate; however, such a high agitation rate may cause severe vortexing and strong rotational flows that disrupt normal DO and *p*CO_2_ control.

**FIGURE 3:**
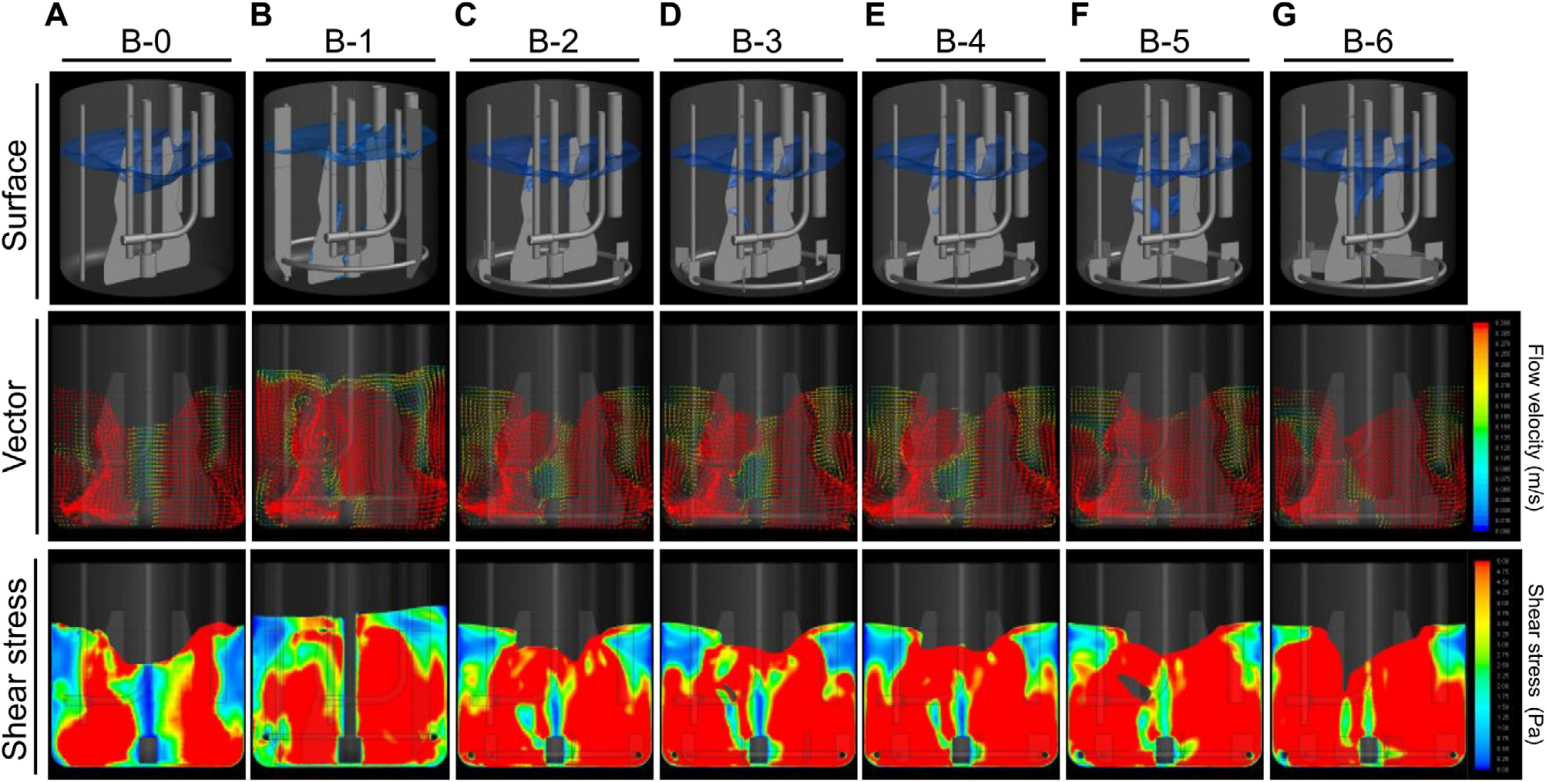
Design of SSDMs and hydrodynamic pattern simulated by CFD.

Here, we developed an SSDM that can reproduce high average shear stress at a lower tip speed without vortex formation or gas hold‐up. Figure 3 shows the seven investigated vessel designs and images of the gas–liquid surface, flow vector, and shear stress at a tip‐speed of 0.97 m/s analyzed by the VOF method. The results of CFD analysis indicated that the average shear stress of the B‐0 was 4.28 Pa, and that this was not sufficient to reproduce the shear stress of manufacturing‐scale BRs. We then focused on an impeller and baffle design based on the B‐0 to obtain an ideal configuration for generating high average shear stress. Of note, the 3 L GBR‐2 and ‐3 had the same configuration as the B‐1 and B‐6, respectively.

First, the effect of three baffles was evaluated. Comparison of the B‐1 with the B‐0 indicated that the baffles help increase hydrodynamic stress; for example, average shear stress was increased by 1.77 Pa (B‐0: 4.28 Pa, B‐1: 6.05 Pa). However, simulation showed that the baffles caused severe gas hold‐up at a tip‐speed of 0.97 m/s (Figure 3A,B). Second, the stationary blades installed at the bottom of the B‐0 were 80% shorter than the baffles of the B‐1. These short‐length baffles were expected to have little impact on flow at the liquid surface but to generate high shear stress near the blades. The B‐2 and B‐3 were equipped with six and eight stationary blades, respectively. CFD analysis revealed that shear stress was higher at the clearance between the impeller and the stationary blades (data not shown). Although severe gas hold‐up did not occur, average shear stress of the B‐2 and B‐3 (B‐2, 5.75 Pa; B‐3, 5.90 Pa) were found to be slightly lower than that of the B‐1, so additional modifications were needed to generate substantially higher shear stress. We, therefore, used wider stationary blades than those before simulation as the B‐4, in which clearance from the stationary blades to the impeller edges was only 3 mm. The CFD results showed that the wider stationary blades increased average shear stress by 0.62 Pa (B‐4: 6.37 Pa) from the B‐2. Another internal study suggested that the number of supplementary impellers was more important to generating shear stress than the number of stationary blades installed (data not shown). Third, supplemental impellers were added to the impeller of the B‐4 and employed CFD analysis (B‐5, two supplemental impellers added; B‐6, four supplemental impellers added; i.e., the 3 L GBR‐3). The data indicated that supplemental impellers generate much higher shear, and that the average shear stress of the B‐6 (9.20 Pa) was 1.5 times higher than that of the B‐1.

**FIGURE 4:**
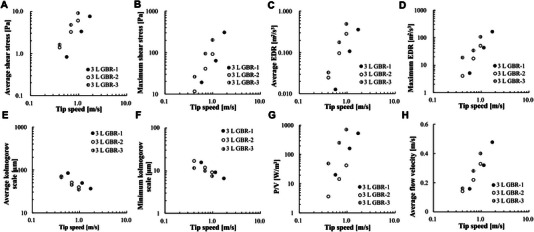
3 L GBR information: regression between CFD output and tip‐speed. EDR indicates energy dissipation rate; GBR, glass bioreactor; *P*/*V*, power input per volume.

**FIGURE 5:**
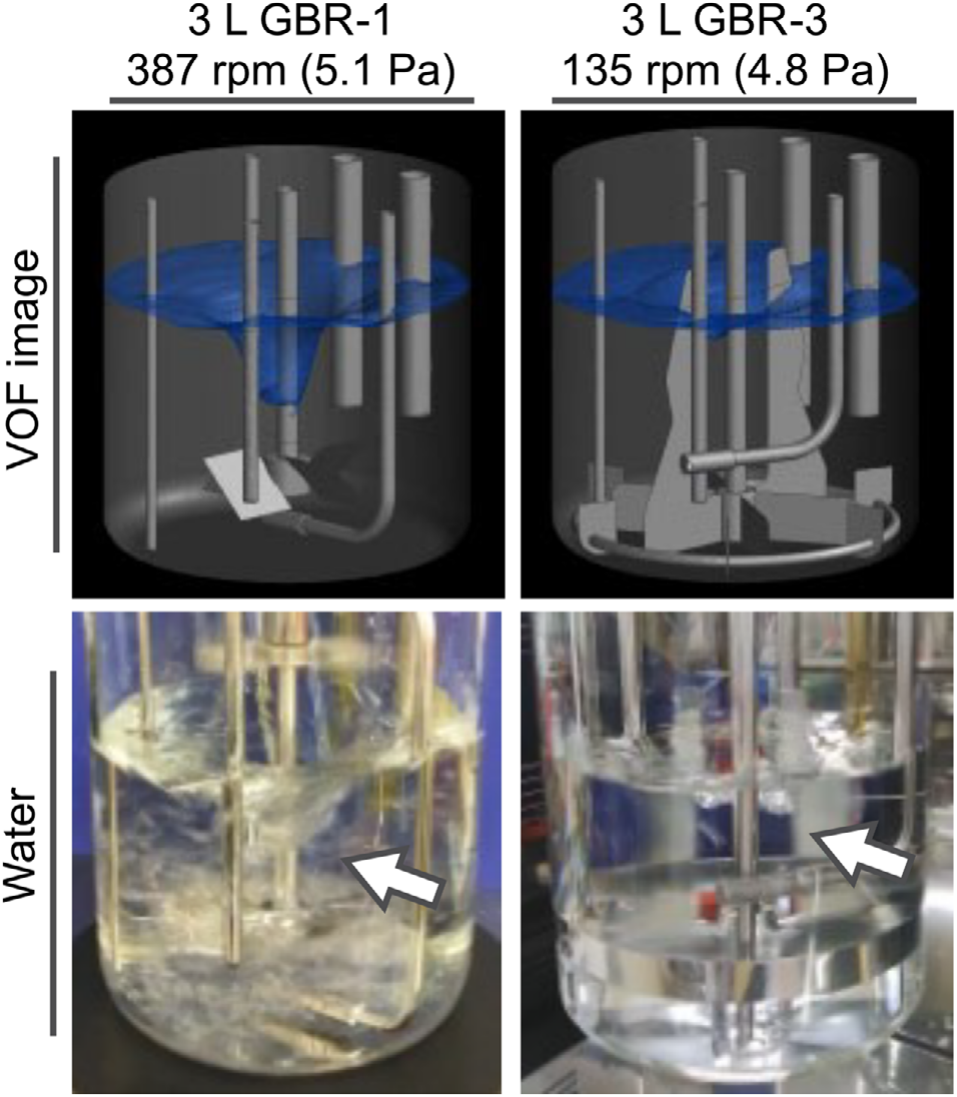
3 L GBR‐3 information: images of the liquid surface. GBR indicates glass bioreactor; VOF, volume of fluid.

Figure 4 shows log–log plots between CFD output and tip‐speed of the 3 L GBR‐1, ‐2, and ‐3. The results indicate that the 3 L GBR‐3 can reproduce the shear stress that covers the shear environment of the manufacturing‐scale BRs at up to a 5 kL scale and at a lower agitation rate. We confirmed that the 3 L GBR‐3 did not form a vortex to generate an average shear stress of 4.8 Pa at least (Figure 5). We, therefore, concluded that the 3 L GBR‐3 can be used as an SSDM to evaluate high shear stress conditions on cells under DO‐ and *p*CO_2_‐controlled conditions.

### 3.3 Understanding the Threshold of High Shear Stress Impact

Using the results of the CFD analysis, we evaluated whether there was a good correlation between cell culture performance and BR parameters. First, fed‐batch culture was performed at several agitation rates using BRs with various scales and configurations. Second, multivariate analysis by CFD output and culture results was performed to identify the correlation. Finally, the threshold of cell damage was evaluated in detail using cell line A as a model cell line. The results are described in the following sections.

### 3.4 Cell Culture Trends

**FIGURE 6:**
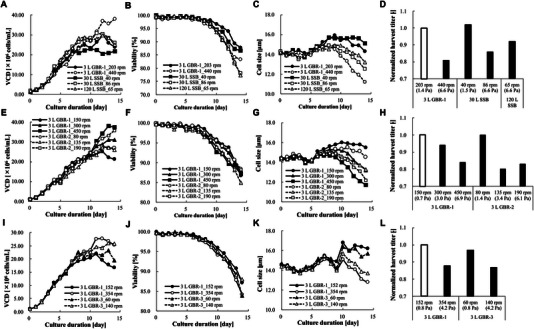
Cell culture profiles under several agitation conditions. GBR indicates glass bioreactor; SSB, stainless steel bioreactor; VCD, viable cell density.

To compare culture performance at different scales and configurations, cell line A was cultured using the 3 L GBR‐1 and the 30 and 120 L SSBs. Figure 6A–C shows the culture profiles at the three different scales. Agitation rates of the 3 L GBR‐1 and 30 L SSB were determined based on the average shear stress matching scale‐up criterion. The agitation rate of the 120 L SSB was based on the same theory to meet the higher shear stress of the other scales. The scale‐independent process parameters were set as the same across the scales. *p*CO_2_ was controlled below 60 mmHg throughout the fed‐batch culture since cell line A was sensitive to high *p*CO_2_. A lower agitation rate can cause excessive aeration due to low kLa, which negatively impacts culture performance. To evaluate the impact of a lower agitation rate on the 30 L SSB, the effect of the same aeration (vvm) through the same type of sparger was evaluated in the 3 L GBR‐1 in another study and found no impact on culture performance (data not shown). To exclude any differences before inoculation, seed culture derived from the same subculture was used for all conditions except for the 30 L SSB at an agitation rate of 40 rpm. The culture profiles suggested that cell culture was successfully scaled‐up based on the same average shear stress. Further, VCD, viability, cell size, and titer were varied by agitation rate. Results showed that the average cell size became smaller from the mid culture phase under high average shear stress conditions across scales (Figure 6C). Harvest viability and titer at the higher shear stress conditions were lower than at the lower shear stress conditions (Figure 6B,D), indicating that higher shear stress induces cell damage and has a negative, albeit not lethal, impact on culture performance. The trends of lactate and ammonium were generally consistent between the conditions; however, under high shear conditions, a slight decrease in lactate consumption and a reduction in ammonia accumulation were observed from the mid culture phase (Figure S1). Of note, the “measured” VCD was consistently higher at the higher average shear stress condition across all scales and configurations (Figure 6A) because fragments smaller than CHO cells emerged from the mid culture phase and were randomly counted as viable cells using a ViCELL XR.

To further investigate the relationship between agitation rate and CHO culture performance, another 3 L GBR‐1, ‐2, and ‐3 run was carried out for cell line A. Figure 6E–H shows the culture profiles using the 3 L GBR‐1 and ‐2, with agitation rates set at three levels for each. The culture trends suggested that the higher tip‐speed, the lower the trends of viability, cell size, and titer. Figure 6I–L shows the culture results using the 3 L GBR‐1 and ‐3 with another seed culture. Agitation rates were set at two levels for both based on the average shear stress matching criterion (0.8 and 4.2 Pa). Culture trends were found to be quite similar among the same average shear stress conditions. As with the results above, lower viability, cell size, and titer were observed at higher average shear stress conditions.

Considering that cell fragments were observed from the mid culture phase onwards under high shear conditions, we suggested that physical cell membrane damage is the primary trigger for the observed reduction in titer, cell size, and viability. Although no significant slowdown in cell growth or abnormal metabolites trends (i.e., lactate and ammonium) were observed, other hypotheses, such as the potential contribution of metabolic disorders or other stress‐related pathways, are also considered. Several studies have attempted to reveal the molecular response of CHO cells to hydrodynamic damage, for instance, through transcriptome analysis [34, 35]. A study focusing on the cellular response of HEK293 to shear stress showed that high shear stress can lead to apoptosis through three pathways (i.e., endoplasmic reticulum stress, cytoskeleton reorganization, and extrinsic signaling pathways) [36]. The application of these approaches may provide insight into the details of the cellular response to shear stress in this case.

### 3.5 Multivariate Analysis

A multivariate analysis was conducted to identify correlations between parameters reflecting the culture environment and titer as an indicator of culture performance. Among the 10 parameters analyzed, average shear stress had the highest correlation with product titer (*ρ* = −0.64) (Table 2). This result supports a previous study, which found that maximum shear stress did not correlate well with cell culture performance, because the region where maximum shear is generated is markedly limited, and cells have little chance to pass through it [37]. Another study suggested that it is also necessary to consider the distribution of shear stress in the BR or the frequency of passage through the high hydrodynamic stress region [17, 38, 39]. Li et al. proposed that the three‐dimensional shear space, consisting of the average of shear strain rate in the impeller zone, tank zone, and whole zone, could be used to identify suitable operating ranges for scale‐up of Sf9 insect cells [40]. Kreitmayer et al. suggested that the impeller zone average strain rate is a scale‐up criterion in geometrically dissimilar SUBs [41].

**TABLE 2:** Correlation between CFD output and product titer.

Parameters	Agitation rate (rpm)	Tip‐speed (m/s)	Shear stress (Pa)		EDR (m^2^/s^3^)		Kolmogorov scale (µm)		P/V (W/m^3^)	Flow velocity (m/s)
			Average	Max	Average	Max	Average	Min		Average
*ρ*	−0.25	−0.47	−0.64	−0.58	−0.34	−0.44	0.53	0.49	−0.47	−0.45
*p* value	0.1215	< 0.01	< 0.0001	< 0.0001	< 0.05	< 0.01	< 0.001	< 0.01	< 0.01	< 0.01

Abbreviations: *ρ*, correlation coefficient; EDR, energy dissipation rate; *P*/*V*, power input per volume.

In our present study using the dry‐wet approach, we found that the average shear stress in a BR is a sufficient indicator of the shear environment under “normal” operating conditions for CHO cells. Importantly, from a practical point of view, it is simply and easily calculated. We, therefore, suggest that average shear stress is a useful factor associated with agitation, and that it can be used for scale‐up, scale‐down, and BR changes among dissimilar configurations.

Regarding the Kolmogorov scale, the average values calculated using the average EDR were consistently much larger than the measured cell size (Figures 2E and 6C,G,K), which may have led to a poor correlation with culture performance. Recently, Freiberger et al. suggested that the Kolmogorov scale is not a suitable indicator of hydrodynamic damage in suspension cell cultures because no major correlation was found between the Kolmogorov scale and culture performance in their system [42]. The validity of the Kolmogorov scale in suspension culture may require further investigation.

### 3.6 Quantification of Shear Stress That Has a Negative Impact on Titer

The threshold of average shear stress that caused a titer decrease in cell line A was evaluated. Titer at harvest (typically Day 14 from inoculation) was used as an indicator of culture performance. Figure 6D,H,L shows the titer on Day 14 under each culture condition described in Section 3.1. Compared with the control conditions, namely the 3 L GBR‐1 at 203 rpm (1.4 Pa), no titer difference was observed with the 30 L SSB at 40 rpm (1.5 Pa). In contrast, the 3 L GBR‐1 at 440 rpm (6.6 Pa) caused a titer reduction of 19%. The same degree of decrease was seen at the 30 L SSB at 86 rpm (6.6 Pa). For the 120 L SSB at 65 rpm (6.6 Pa), viability and cell size were apparently decreased; although the decrease in titer was less than expected, and less than that with the 30 L SSB at 86 rpm (Figure 6D), it was nevertheless within the range of cultural and analytical variability. Figure 6H shows the titer of 3 L GBR‐1 and ‐2 and suggests that the threshold of average shear stress associated with a ≥ 10% titer reduction was around 3.0–3.4 Pa. This outcome was supported by the results in Figure 6L, in which almost the same degree of titer decrease was observed under the higher agitation rate conditions compared to the standard conditions. Taken altogether, these findings indicate that cell line A has a threshold of titer reduction due to agitation‐associated stress, and that this threshold is well explained by the average shear stress in the BR. Since these results were obtained using an established SSDM (3 L GBR‐3), we consider that we were able to evaluate the effects of hydrodynamic stress due to agitation without the possible influence of the secondary effects peculiar to high agitation rate conditions (e.g., vortex formation, severe gas hold up, and unstable DO and *p*CO_2_ control).

## 4 Application of the Shear Stress Criterion for Clone Selection

Achieving a secure scale‐up requires not only the appropriate determination of operating parameters, but also the selection of a cell line that is resistant to the shear environment. We evaluated the sensitivity of 6 mAb A‐expressing clones and 12 Fab A‐expressing clones to the shear stress caused by higher agitation. All 18 clones were cultured using the 3 L GBR‐1 at 295 rpm (2.9 Pa) unless otherwise noted, and 5 were further cultured under the strengthened stress condition of 394 rpm (5.2 Pa). Table 3 shows the relative ratio of titers under the higher shear‐stress condition to the control on Day 14 (3 L GBR‐1, 150 rpm, 0.7 Pa).

**TABLE 3:** **Response to shear stress among cell lines**.

mAb A‐expressing cell line						
Clone ID	Clone A	Clone B	Clone C	Clone D	Clone E	Clone F
2.9 (Pa)	0.85	1.00	0.87	1.02	1.03^a^	0.93
5.2 (Pa)	0.85	−	0.85	−	−	0.80

Fab A‐expressing cell line												
Clone ID	Clone a	Clone b	Clone c	Clone d	Clone e	Clone f	Clone g	Clone h	Clone i	Clone j	Clone k	Clone l
2.9 (Pa)	1.01	0.98	0.90	1.00	1.01	0.96	0.96	0.85	0.90	0.90	0.86^b^	0.92
5.2 (Pa)	0.97	−	−	−	0.94	−	−	−	−	−	−	−

*Note:* Relative ratio of product titer under the higher shear stress condition versus 0.7 Pa of shear stress as a control.

Abbreviations: Fab, fragment antibody; mAb, monoclonal antibody.

^a^ Evaluated at 3.2 Pa of the average shear stress.

^b^ Evaluated the product titer on Day 12.

In mAb A‐expressing cell lines, a titer reduction of ≥ 10% was observed with two of six clones under an average shear stress of 2.9 Pa. These two clones (clone A and C) and clone F were also evaluated at the average shear stress of 5.2 Pa, and a further titer decrease was confirmed. In Fab A‐expressing cell lines, a titer reduction of ≥ 10% was observed with 5 of 12 clones under an average shear stress of 2.9 Pa. Two clones (clone a and e) were also cultured at an average shear stress of 5.2 Pa, but their titers were only slightly decreased (3% and 6%, respectively). These findings indicate that the threshold of cell damage of mAb A‐ or Fab A‐expressing cell lines was also dependent on individual cell lines.

The average shear stress of manufacturing‐scale BRs at a typical agitation rate, including 1–2 kL SUBs, was estimated to be up to 6 Pa by CFD analysis. In this experiment, the average shear stress of 5.2 Pa resulted in a decreasing titer by ≥ 10% at least 50% of mAb A‐expressing clones and 42% of Fab A‐expressing clones. Although the average shear stress below 2.9 Pa could be achieved at manufacturing‐scale BRs by setting a lower agitation rate and/or modifying the BR design, the undesirable lower agitation rate might cause other critical problems, such as inhomogeneous mixing and a low kLa.

## 5 Concluding Remarks

The aim of this study was to identify a factor in CHO cell culture that reflects hydrodynamic stress and can be used in dissimilar BR scales and configurations. Using the dry‐wet approach, we developed an SSDM (3 L GBR‐3) to evaluate the shear environment of manufacturing‐scale BRs without any secondary effects. Using several BRs of various scales and configurations, we found that average shear stress can be used as a factor to determine the agitation rate required to maintain a consistent product titer among BRs. Further, we suggest that the threshold of titer reduction, as an indicator of cell damage, differs among cell lines. Thus, evaluating the risk of shear sensitivity during clone selection, especially before scaling‐up, is a reasonable strategy to adopt in implementing a relatively uninterrupted scale‐up. Of note, a couple of in‐house cell lines were evaluated at high average shear stress conditions using lab‐scale BRs and were successfully scaled‐up to several 1 and 2 kL SUBs.

Scale‐down from manufacturing‐scale BRs to lab‐scale BRs is also important when understanding and addressing issues occurring with manufacturing‐scale BRs. If a BR needs to be switched to another one during a product lifecycle, cell culture performance should be consistent between BRs. Our findings provide useful tools for securing scale‐up, scale‐down, and cell culture BR changes for the biopharmaceutical industry. Although it is still unclear whether the results of this study are applicable to other CHO strains and cell lines, the concept that average shear stress can summarize the stress environment of a BR and can be applied to the determination of an agitation rate across scales and types of BRs is possibly considered to be a general perspective. In addition, the impact of the average shear stress on product quality, such as aggregates and charge variants, and the deeper mechanism by which shear stress induces the reduction in cell size, viability, and titer need to be analyzed in further studies.

## Author Contributions

**Hiroyuki Kenmoku:** conceptualization, experiments, analysis, draft preparation. **Takanobu Saito:** experiments, analysis, and review. **Shunsuke Ohira:** resources, review, supervision, and project administration. **Akira Kaneko:** CFD simulation, analysis, and review. **Takahiro Nemoto:** CFD analysis and review. **Yoshikazu Kato:** CFD analysis and review.

## Conflicts of Interest

The authors declare no conflicts of interest.

## Supporting information




**Supporting File 1:** elsc70054‐sup‐0001‐SuppMat.pdf

## Data Availability

The data that support the findings of this study are available from the corresponding author upon reasonable request.
