# Supplementary material for: Scale‐Up Strategy Focused on Hydrodynamic Stress for Mammalian Cell Culture Established by a Dry‐Wet Approach
Source: Eng Life Sci. 2025 Nov 21;25(11):e70054. doi: 10.1002/elsc.70054 (PMC12635869; doi:10.1002/elsc.70054)
Supplement: Supplementary file 1 — Supporting File 1: elsc70054‐sup‐0001‐SuppMat.pdf [file ELSC-25-e70054-s001.pdf]

Supporting information

Figure S1 The trends of lactate and ammonium

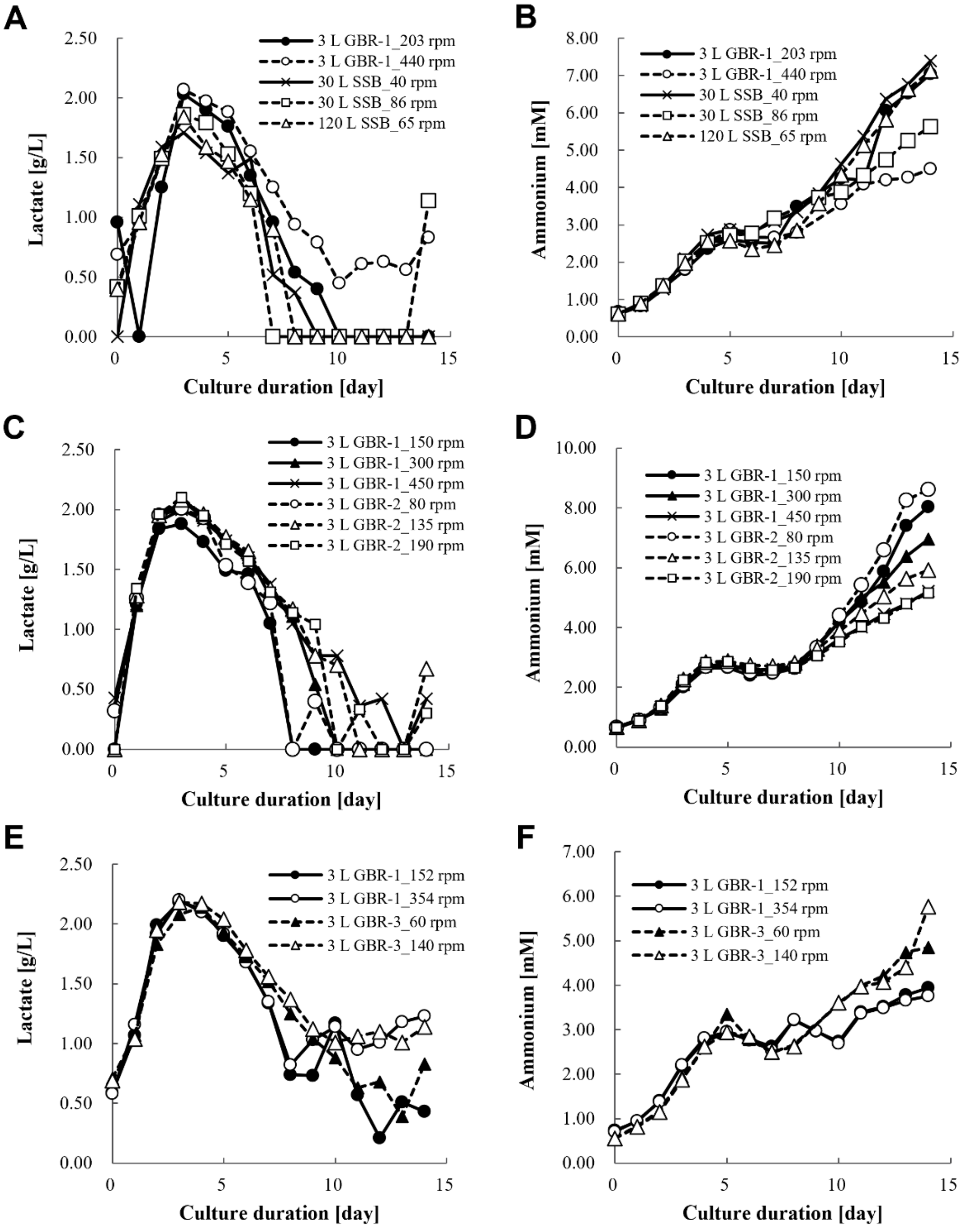

Abbreviations: GBR, glass bioreactor; SSB, stainless steel bioreactor
